# Pyrosequencing *Bacillus anthracis*

**DOI:** 10.3201/eid1110.041316

**Published:** 2005-10

**Authors:** Tara Wahab, Sandra Hjalmarsson, Ralfh Wollin, Lars Engstrand

**Affiliations:** * Swedish Institute for Infectious Disease Control, Solna, Sweden

**Keywords:** Bacillus anthracis, species verification, pyrosequencing, rpoB, research

## Abstract

Pyrosequencing technology is a sequencing method that screens DNA nucleotide incorporation in real time. A set of coupled enzymatic reactions, together with bioluminescence, detects incorporated nucleotides in the form of light pulses, which produces a profile of characteristic peaks in a pyrogram. We used this technology to identify the warfare agent *Bacillus anthracis* by sequencing 4 single nucleotide polymorphisms (SNPs) in the *rpoB* gene as chromosomal markers for *B. anthracis*. In addition, 1 segment in each of the *B. anthracis* plasmids pXO1 and pXO2 was analyzed to determine the virulence status of the bacterial strains. Pyrosequencing technology is a powerful method to identify *B. anthracis*.

## Abstract 2

Pyrosequencing technology was used to rapidly and specifically identify *Bacillus anthracis.*

*Bacillus anthracis* is a gram-positive, rod-shaped, spore-forming bacterium that causes the acute mammalian disease anthrax when endospores enter the body. The infection is often fatal if not treated with antimicrobial drugs before or when the first symptoms appear. The bacterium can infect livestock and humans by gastrointestinal, cutaneous, or respiratory routes. Potentially, *B. anthracis* spores can be an effective biological weapon because of their high stability. They do not divide, have no metabolism, and are resistant to drying, heat, UV light, and many disinfectants. In 2001, letters containing anthrax spores caused illness in 22 persons, leading to 5 deaths in the United States.

*B. anthracis* has 2 plasmids: the toxin-encoding pXO1 (182 kb) and capsule-encoding pXO2 (95 kb) (*1**,**2*). Both plasmids are required for virulence; lacking 1 of the plasmids attenuates the microorganism. The pXO1 plasmid contains genes *lef*, *cya*, and *pag*, which encode the toxin's lethal factor, edema factor, and protective antigen, respectively (*3**–**5*). The pXO2 plasmid contains the genes *capA*, *capB*, and *capC*, necessary for capsule formation (*6*). These genes have been used as markers to identify *B. anthracis* with polymerase chain reaction (PCR) in both environmental and clinical samples (*7**–**9*).

Differentiating between *B. anthracis* and closely related *B. cereus* and *B. thuringiensis* is difficult (*10*). Usually, phenotypic characteristics, such as susceptibility to β-lactam antimicrobial drugs, lack of hemolysis, lack of motility on sheep blood agar plate, and inability to ferment salicin, are used to differentiate (*11**,**12*). A variety of chromosomal markers that appear to be specific have been suggested for genotypic species determination of *B. anthracis* (*13**–**18*). We studied single nucleotide polymorphisms (SNPs) in the *rpoB* gene, described by Qi et al. (*13*) by using pyrosequencing technology (*19*). This technology can determine SNPs and short DNA stretches in real time, starting from PCR products. Biotinylated PCR amplicons that cover the region of interest are immobilized onto solid streptavidin coated beads and converted to single-stranded form. A sequencing primer is hybridized to the single-stranded DNA, and incorporation of added nucleotides is detected as light peaks by an enzymatic cascade. Enzymatic degradation of excess nucleotides allows the reaction to be performed in a single tube. When one starts from PCR products, <96 genetic targets can be sequenced within 1 hour.

In this study, we used the *rpoB* gene as a chromosomal marker to discriminate between *B. anthracis* and closely related bacillus species. We studied 4 *B. anthracis*–specific *rpoB* SNPs located at positions 911, 912, 913, and 914 in duplex sequencing reactions by using a unique sequencing primer for each desired SNP in a collection of 17 anthracis and 10 non-anthracis *Bacillus* strains. Simultaneously, we investigated the distribution of virulence plasmids pXO1 and pXO2 among these strains by using PCR and pyrosequencing technology to rapidly verify the amplicons.

## Materials and Methods

The *B. anthracis* reference strains used in this study were obtained from the National Collection of Type Cultures, London, England, and the Swedish Defense Research Agency. Reference strains of *B. cereus*, *B. mycoides*, and *B. thuringiensis* were obtained from the Culture Collection University of Gothenburg of Sweden. All bacterial strains are listed in Table 1. Bacteria were cultured on blood sheep agar at 37°C for 16 h, and genomic DNA was prepared by using a commercially available DNA extraction kit, QIAamp tissue protocol (Hilden, Stockholm, Sweden). The DNA was boiled at 99°C for 15 min, plated on blood agar, and incubated for 3 days. No growth was observed, and the DNA was removed from the biosafety level 3 laboratory. All material, including the DNA, is under the protection of our institute.

**Table 1:** Bacterial strains and their plasmid content*

Strain	Origin	pXO1	pXO2
*Bacillus anthracis*			
30	UK	+	+
57-77	UK	+	+
73-77	UK	+	+
183-78	UK	+	+
187-78	UK	+	+
188-78	UK	+	+
R81/04	Iceland	+	+
NCTC 10340	Unk.	+	+
NCTC 2620	UK	+	+
NCTC 109	UK	+	–
NCTC 1328	UK	+	–
NCTC 5444	UK	–	+
NCTC 7752	UK	–	+
NCTC 7753	UK	–	+
NCTC 08234	UK (Sterne)	+	–
ATCC 4229	Unk.	–	+
Sterne 7702	UK	+	–
*B. cereus*			
CCUG 7414	USA	–	–
CCUG 10781	USA	–	–
CCUG 36925	USA	–	–
CCUG 41923	Sweden	–	–
CCUG 43518A	Sweden	–	–
CCUG 46981	Sweden	–	–
CCUG 47340	UK	–	–
*B. mycoides*			
CCUG 26678	USA	–	–
*B. thuringiensis*			
CCUG 7429	Germany	–	–
CCUG 22499	UK	–	–

*Unk., unknown; NCTC, National Collection of Type Cultures; CCUG, Culture Collection University of Gothenburg.

### PCR

All reagents used for amplification of bacterial DNA were from Amersham Biosciences (Uppsala, Sweden) except for primers, which were from Invitrogen Life Technologies (Paisley, United Kingdom). Table 2 shows the primer sequences. The reverse primer for each PCR fragment was biotinylated. PCR primers were designed to amplify a 176-bp fragment of *rpoB*, 179 bp of the pXO1 plasmid, and 127 bp of the pXO2 plasmid. PCR was performed in 50-μL reaction mixtures containing 1× PCR buffer (10 mmol/L Tris-HCl, pH 8.3, 50 mmol/L KCl, 2.5 mmol/L MgCl_2_), 0.8 U Taq DNA polymerase, 0.2 mmol/L each nucleotide, 0.1 μmol/L each primer and 5 μL eluate containing DNA. The reaction mixture was subjected to 95°C for 5 min and 45 cycles of 95°C for 30 s, annealing at 60°C for 30 s, and elongation at 72°C for 30 s, followed by terminal extension at 72°C for 7 min.

**Table 2:** Sequence of primers used in PCR and sequencing*

Target	Primer	Sequence
*rpoB*	PCR forward	5´-CCACCAACAGTAGAAAATGCCA-3´
	PCR reverse	b-5´-AAATTTCACCAGTTTCTGGATCTA-3´
	Sequencing 911	5´-CACCAACAGTAGAAAATGC-3´
	Sequencing 912	5´-TTCTTCGATCCAAAGC-3´
	Sequencing 913	5´-TAAAAACAGATTGTTTA-3´
	Sequencing 914	5´-AACGTTTAGCTGAAACATT-3´
pXO1	PCR forward	5´-GATATGAACCCGTACTTGTAAT-3´
	PCR reverse	b-5´-GAATCAGATGCATTTTTAATGG-3´
	Sequencing	5´-TTTATTATGAAATAGGT-3´
pXO2	PCR forward	5´-TTCGCCTTGTGATTCCGCACT-3´
	PCR reverse	b-5´-GGGCGCTGGTCGTATGTTTAG-3´
	Sequencing	5´- TGTTTGTCCACTTCC-3´

*PCR, polymerase chain reaction; b, biotin.

### Pyrosequencing Analysis

Two primers were designed to sequence of 30 nucleotides within the PCR amplicons generated from plasmids pXO1 and pXO2, respectively. In addition, 1 sequencing primer was designed for each of the 4 *rpoB* SNPs to be determined (Table 2). The *rpoB* primers were used in duplex sequencing reactions so that primers 911 and 912 were combined in a single reaction for sequencing of SNPs at positions 911 and 912, while primers 913 and 914 were used for combined sequencing of SNPs at positions 913 and 914. For sequencing according to the pyrosequencing technology, biotinylated PCR amplicons were immobilized onto streptavidin-coated magnetic beads and denatured to produce single-stranded DNA by using a PSQ 96 Sample Prep Tool (Biotage AB, Uppsala, Sweden). Sequencing primers were added and allowed to hybridize to the strands, after which sequencing was performed according to the manufacturer's instructions. All steps were performed at room temperature.

## Results

### Bacterial Strains and DNA Extraction

*B. anthracis* was unambiguously identified by determining 4 SNPs in the *rpoB* gene by using pyrosequencing technology; thus, we were able to distinguish *B. anthracis* from the other tested bacillus species. The Figure shows representative output diagrams, or pyrograms, from duplex sequencing reactions of the SNPs in *B. anthracis* National Collection of Type Cultures 2026 and *B. cereus* Culture Collection University of Gothenburg 7414. The specific nucleotides 911C, 912T, 913C, and 914A were found in all *B. anthracis* strains tested and appear to be unique to *B. anthracis.* The corresponding nucleotides in all tested non-anthracis strains (*B. cereus*, *B. thuringiensis*, and *B. mycoides*) were 911T, 912C, 913T, and 914G. In duplex sequencing reactions, we could easily determine 2 SNPs in each pyrogram (Figure).

**Figure:**
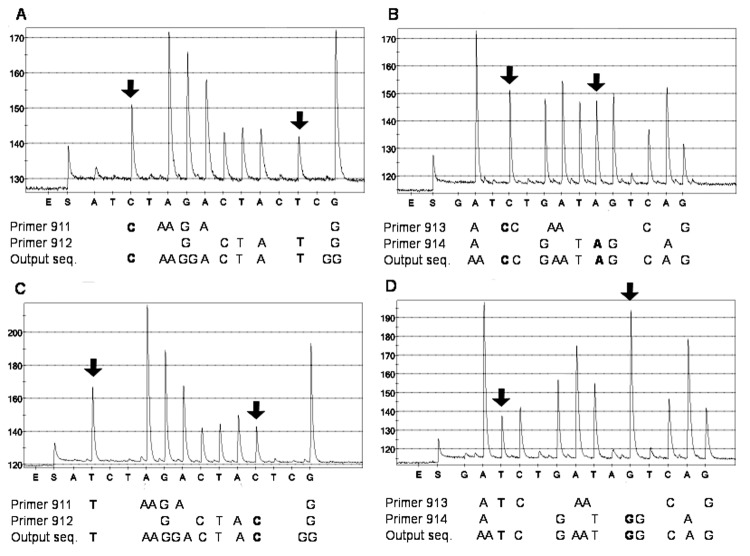
Sequence analysis of single nucleotide polymorphisms (SNPs) in the rpoB gene of Bacillus anthracis National Collection of Type Cultures (NCTC) 2026 (A and B) and B. cereus Culture Collection University of Gothenburg (CCUG) 7414 (C and D). The initial addition of enzyme (E) and substrate (S) mixture and the sequential addition of nucleotides are shown on the x axis. An initial peak was generated when the substrate mixture was added due to pyrophosphate molecules remaining from the polymerase chain reaction (PCR) reaction. Incorporation of added nucleotides was detected as light peaks, measured in relative light units as shown on the y axis. Incorporation of 2 identical consecutive nucleotides gave rise to peaks that were higher than those generated when a single nucleotide was incorporated. The 4 SNPs were determined by using the 4 sequencing primers 911–914 in 2 duplex reactions per strain. The output sequence reported by the software was a combination of the 2 unique sequences produced by each primer in the duplex reactions. The SNPs distinguishing B. anthracis NCTC 2026 from B. cereus CCUG 7414 are shown in bold.

Presence of the virulence plasmids was determined by PCR and sequence verification of regions on plasmids pXO1 and pXO2 (Table 1). The assay was applied on 17 *B. anthracis* strains, 7 *B. cereus* strains, 2 *B. thuringiensis* strains, and 1 *B. mycoides* strain. Of the 17 *B. anthracis* isolates investigated, 9 isolates were positive for both pXO1 and pXO2, while the remaining 8 lacked either 1 or both of the virulence plasmids. All non-anthracis strains were negative for both plasmids. pXO1 and pXO2 PCR amplicons originating from the *B. anthracis* strains were verified by sequencing 30 nucleotides with the pyrosequencing technology. The nucleotide sequence following the sequencing primers located within the amplified fragments was AAGATATTATCAAGGGATATTTTAAGTAAA for all pXO1 amplicons and ACCACTCATTAAGTTCTTCGCACCGCTAAA for all pXO2 amplicons, which agreed with all nucleotide sequences of these regions submitted to the GenBank nucleotide database (http://www.ncbi.nlm.nih.gov), accession numbers AF065404, AE011190, AJ413934, AE017336, AJ413935, M29081, M30210, AF188935, AE011191, and AE017335. *B. anthracis* was successfully identified by using pyrosequencing technology for genotyping 4 SNP positions of the *rpoB* gene, which appear to be specific for *B. anthracis*, and 2 fragments of virulence plasmids pXO1 and pXO2.

## Discussion

In biologic warfare, speed and accuracy are in high demand for identifying and characterizing microbial species. In this study, we investigated the possibility of using pyrosequencing technology to rapidly identify and characterize strains of *B. anthracis* and distinguish them from related non-anthracis *Bacillus* strains. This method has been used to analyze multiple targets that are important in microbial infections (*20**,**21*).

By determining 4 SNPs in the *rpoB* gene, *B. anthracis* strains were successfully identified. This chromosomal marker can discriminate between *B. anthracis* and other closely related species from the *Bacillus* genus. The 16S rRNA gene cannot be relied upon to differentiate *B. anthracis* from its close relatives; therefore, we did not include this target in the assay (*22*). By careful design of nucleotide dispensation order, multiple SNPs may be analyzed in 1 single sequencing reaction by using a unique primer for each desired SNP. To save time and reduce reagent cost, we analyzed the *rpoB* SNPs in duplex pyrosequencing reactions. The resulting pyrograms of overlapping sequences were easily resolved by the accompanying software (Figure). Using 1 well for all 4 SNP positions may further optimize the method.

This technology validates PCR-based assays by qualitatively verifying that a positive PCR result is not the effect of nonspecific amplification, as shown here by sequence verification of PCR amplicon generated from virulence plasmids pXO1 and pXO2. The risk of false-positive results is thereby minimized.

We illustrate for the first time how pyrosequencing technology can identify *B. anthracis*. Using this technology in diagnostic laboratories is advantageous because it is rapid, simple, nonradioactive, inexpensive, and automated. It is a powerful method to rapidly determine genetic targets; as many as 96 samples can be analyzed in 40 minutes. Genetic analysis with pyrosequencing technology could make selecting antimicrobial drug treatment easier and potentially complement typing methods and time-consuming, traditional microbial identification, such as biochemical testing, phage lysing assays, and immunologic assays.
